# First-Principles Calculations of Plasmon-Induced Hot Carrier Properties of μ-Ag_3_Al

**DOI:** 10.3390/nano15100761

**Published:** 2025-05-19

**Authors:** Zihan Zhao, Hai Ren, Yucheng Wang, Xiangchao Ma, Jiali Jiang, Linfang Wei, Delian Liu

**Affiliations:** 1 School of Optoelectronic Engineering, Xidian University, Xi’an 710071, China; xd_zzh@stu.xidian.edu.cn (Z.Z.); jiali2716@163.com (J.J.); weilinfang@xidian.edu.cn (L.W.); dlliu@xidian.edu.cn (D.L.); 2 Shanghai Aerospace Institute of Electronic Technology, Shanghai 201109, China; renhai92@126.com

**Keywords:** μ-Ag_3_Al, surface plasmon, non-radiative decay, carriers transport

## Abstract

Non-radiative decay of surface plasmon (SP) offers a novel paradigm for efficient conversion of photons into carriers. However, the narrow bandwidth of SP has been a significant obstacle to the widespread applications. Previously, research and applications mainly focused on noble metals such as Au, Ag, and Cu. In this article, we report an Ag-Al alloy material, μ-Ag_3_Al, in which the surface plasmon operating bandwidth is 1.7 times that of Ag and hot carrier transport properties are comparable with those of AuAl. The results show that μ-Ag_3_Al allows efficient direct interband electronic transitions from ultraviolet (UV) to near infrared range. Spherical nanoparticles of μ-Ag_3_Al exhibit the localized surface plasmon resonance (LSPR) effect in the ultraviolet region. Its surface plasmon polariton (SPP) shows strong non-radiative decay at 3.36 eV, which is favorable for the generation of high-energy hot carriers. In addition, the penetration depth of SPP in μ-Ag_3_Al remains high across the UV to the near-infrared range. Moreover, the transport properties of hot carriers in μ-Ag_3_Al are comparable with those in Al, borophene and Au-Al intermetallic compounds. These properties can provide guidance for the design of plasmon-based photodetectors, solar cells, and photocatalytic reactors.

## 1. Introduction

Surface plasmons (SPs) are the collective coherent oscillations of electrons at the surface of conductors or heavily doped semiconductors, which, upon excitation, decay through various channels, including radiative and nonradiative decay, thereby converting the light energy into other forms of energy [1,2,3]. Nonradiative decay of SP can generate high-energy carriers, known as hot carriers, with their average energy greater than that of carriers in thermal equilibrium with crystal lattice. From the perspective of SP propagation applications, nonradiative decay is considered as a loss of SP that should be addressed by prolonging the lifetime and/or propagation length of a SP. However, from the perspective of light-harvesting antenna applications, nonradiative decay provides a powerful means to efficiently convert absorbed photons into hot carriers. Recent studies and applications of surface plasmon-induced hot carriers have been reported in the fields of photodetection [4,5], photoelectric conversion [6,7,8], and photocatalysis [9,10].

Surface plasmon polariton (SPP) is electromagnetic excitation arises via the coupling of electromagnetic fields to the SP [11,12,13]. The hot carriers induced by nonradiative decay of SPP can be collected as photocurrents, thereby enhancing the performance of the entire system in applications. For example, Ali Sobhani et al. excited SPP through the gratings, significantly enhancing the responsivity and internal quantum efficiency of the photodetector [14]. Fang Zhou et al. experimentally constructed an Au@ZnO@PS structure and studied the variation of photocurrent with ammonia concentration at room temperature [15]. Kai Yao et al. embedded plasmonic nanostructures Ag@TiO2@Pa in the active layer of solar cells, where hot electrons from the plasmon are extracted to generate a larger photocurrent [16]. Deepak Sharma et al. enhanced the power conversion efficiency of solar cells by incorporating Ag nanoparticles [17].

Most SP applications rely on noble metals (Au, Au, Cu) [18,19,20,21,22,23]. Among them, Au is the preferred choice for commercial plasmonic detectors due to its low surface plasmon (SP) losses and chemical stability under a wide range of conditions. Due to the scarcity of Au, Ag and Cu are commonly used as substitutes. Ag exhibits superior sensitivity and reflectivity compared with Au, and Cu possesses the best electrical conductivity and demonstrates optical properties comparable with those of Au and Ag. However, their applications in this field are limited due to their poor chemical stability. In summary, it is essential to develop inexpensive, stable, and high-performance plasmonic materials. Compared with the corresponding pure metals, alloys can offer superior properties in some cases, so extensive exploration of metal alloys has been conducted [24,25,26,27]. There are reports on the plasmonic applications of Ag-Al alloys. Parashar et al. embedded Ag_2_Al nanoparticles in silicon solar cells, enhancing solar conversion efficiency [28]. Xue-Mei Wen et al. used Ag-Al alloy electrodes and, through the appropriate composition ratio of the Ag-Al alloy, they could effectively excite SPP, thereby improving the brightness and current efficiency of OLEDs [29]. On the other hand, Ag-Al alloys have been widely used in electronic packaging. Notably, Shao-Wei Fu et al. studied the specific formation process of Ag-Al alloys and found that a stable μ-Ag_3_Al phase can form at the Ag-Al interface after prolonged annealing at 200 °C [30]. Kuang-kuo Wang et al. investigated the orientation relationships and interface between μ-Ag_3_Al and Ag, as well as formation sequence of γ-Ag_2_Al and μ-Ag_3_Al at the Ag/Al interface [31].

Although there have been numerous reports on the phase transitions in the Ag-Al binary system and the relevant properties of Ag-Al alloys [32,33,34,35,36], there has been a lack of focus on the optical properties of the Ag-Al alloy with a defined atomic composition and a well-determined space group symmetry. Considering the good chemical stability of μ-Ag_3_Al, in this work, we study its optical, plasmonic, and hot carriers properties. Specifically, we investigate its electronic structure, dielectric function, LSPR, SPP dispersion and loss characteristics, as well as the generation and transport of hot carriers through first-principles calculations. It is found that the energy bands of μ-Ag_3_Al are so densely packed that photons in the infrared region can also excite direct interband transitions. Spherical nanoparticles of it exhibit the LSPR effect in the ultraviolet region. Different from Ag, its SPP can undergo non-radiative decay over a wide spectral range. Moreover, μ-Ag_3_Al is capable of exciting a large number of hot carriers under light irradiation from UV to near-infrared range. Additionally, the transport properties of the hot carriers are comparable to those of Al, borophene, and Au-Al alloy.

## 2. Method

### 2.1. Computational Details

To obtain the electronic structure, phonon spectrum, and electron-phonon matrix elements, we use open-source code JDFTx [37] for the first-principles calculations. To describe the exchange-correlation energy and truncated Coulomb interactions, we used fully relativistic norm-conserving pseudopotentials at the plane-wave cutoff energy of 25 Hartrees and the generalized gradient approximation (GGA) functional of Perdew, Burke and Ernzerhof. During structure optimization and self-consistent ground state calculations, the Brillouin zones for μ-Ag_3_Al are sampled with 6 × 6 × 6 gamma-centered k-points with a Fermi-Dirac smearing of 0.01 Hartrees. Phonon calculations employ a 2 × 2 × 2 supercell for μ-Ag_3_Al. These parameters ensure convergence of the calculation results.

Subsequently, the calculated electron, phonon, and electron-phonon matrix elements are transformed to the maximally-localized Wannier functions (MLWFs) basis representation [38,39]. This allows electronic energy, phonon energy and electron-phonon matrix elements to be obtained at any sampling point in an extremely fine grid. Specifically, we use 122 Wannier basis functions to faithfully reproduce the electronic energy, phonon energy, and electron-phonon matrix elements near the Fermi level. The calculations of the Fermi occupancy of electrons and the Bose occupancy of phonons are performed at a temperature of T = 300 K.

### 2.2. Optical Responses

The optical response of metal materials is mainly influenced by Drude resistance, direct interband, and phonon-assisted intraband electronic transitions. We express the imaginary part of the dielectric function of μ-Ag_3_Al by the frequency-dependent real part of the complex conductivity $\sigma \left(\omega \right)$ [40,41]:

(1) Im⁡εω=Re⁡σωε0ω

The real part of complex conductivity Re⁡σω is calculated as follows [39,40]:

(2) Reσω=σ0τD0−1⋅τD−1ωτD−1ω2+ω2+Reσinterω

where the first term describes the contribution of phonon-assisted intraband electron transitions and Drude resistance, while the second term indicates the contribution of direct interband electron transitions.

To assess the impact of phonon-assisted intraband electron transitions, we calculated the frequency-dependent momentum relaxation rate using the Eliashberg spectral function [42,43]:

(3) $$\tau_{D}^{-1}\left(\omega \right)=\frac{2\pi }{\hbar g\left(\varepsilon_{F}\right)b_{T}\left(\hbar \omega \right)}\sum_{\alpha }\int_{BZ}\frac{dq}{{\left(2\pi \right)}^{3}}\times G_{q\alpha }^{p}b_{T}\left(\hbar \omega -\hbar \omega_{q\alpha }\right)$$

where $\varepsilon_{F}$ is Fermi energy and $g\left(\varepsilon_{F}\right)$ is the density of electronic states near the Fermi level; ℏω is incident light energy; $\hbar \omega_{q\alpha }$ is the energy of phonon with wave-vector ***q*** in mode α; bTε=ε1−e−ε/kBT and the dimensionless $G_{q\alpha }^{p}$ is defined as:

(4) $$\begin{array}{cc} G_{q\alpha }^{p}\equiv & \sum_{nn’}\int_{BZ}\frac{g_{s}\Omega dk}{{\left(2\pi \right)}^{3}}{\left|g_{\left(k+q\right)n’,kn}^{q\alpha }\right|}^{2}\times \left(1-\frac{v_{kn}\cdot v_{\left(k+q\right)n’}}{\left|v_{kn}\right|\left|v_{\left(k+q\right)n’}\right|}\right)\\ & \times \delta \left(\varepsilon_{kn}-\varepsilon_{F}\right)\delta \left(\varepsilon_{\left(k+q\right)n’}-\varepsilon_{F}\right) \end{array}$$

where $\varepsilon_{kn}$ is the energy of electron with wave-vector ***k*** in band **n**; $v_{kn}$ is the band velocity with wave-vector ***k*** in band n; $k_{B}$ is Boltzmann constant, T represents the room temperature and $k_{B}T$ is approximately 0.00094 Hartrees; $g_{\left(k+q\right)n’,kn}^{q\alpha }$ is the electron-phonon matrix element.

To assess the contribution of Drude resistance, the ratio of DC conductivity to the average Drude momentum relaxation time is calculated as follows [44]:

(5) $$\frac{\sigma_{0}}{\tau_{D_{0}}}=\int_{BZ}\frac{e^{2}g_{s}dk}{{\left(2\pi \right)}^{3}}\sum_{n}\delta \left(\varepsilon_{kn}-\varepsilon_{F}\right)\left(v_{kn}⊗v_{kn}\right)$$

The real part of the conductivity due to direct interband transitions is calculated as follow [44]:

(6) $$\begin{array}{cc} {\mathrm{Re\sigma}}_{\mathrm{inter}}\left(\omega \right)= & \varepsilon_{0}\cdot \omega \cdot \frac{\pi e^{2}}{\omega^{2}}\int_{BZ}\frac{g_{s}dk}{{\left(2\pi \right)}^{3}}\sum_{nn’}\left(f_{kn}-f_{kn’}\right)\\ & \times \delta \left(\varepsilon_{kn’}-\varepsilon_{kn}-\hbar \omega \right)(v_{knn’}^{*}⊗v_{knn’}) \end{array}$$

where $f_{kn}$ is Fermi occupation of electrons with wave-vector ***k*** in band n; $v_{knn’}$ is the matrix elements of the velocity operator; $\varepsilon_{0}$ is the vacuum dielectric constant. All terms in the integrand are expressed using Wannier functions for efficient calculation. The real part of the dielectric function is obtained by converting the imaginary part of the dielectric function using the Kramers-Kronig relation [45,46].

For the double integral in the first Brillouin zone in Equation (3), Monte Carlo sampling with 1 × 10^6^ sampling points is used to calculate the frequency-dependent momentum relaxation rate. For Equation (6), the direct interband electron transitions are calculated using Monte Carlo sampling in the Brillouin zone with 8 × 10^4^ sampling points. These parameters ensure the convergence of the calculation results.

### 2.3. Transport Properties

Only the hot carriers that survive long enough and travel a sufficient distance can be effectively collected and detected. Therefore, we characterize the lifetime and mean free path of hot carriers by accounting for the contributions of both electron-electron and electron-phonon scattering.

To evaluate the impact of electron-electron scattering, the imaginary part of the quasiparticle self-energy is calculated as follows [38,47]:

(7) Im⁡∑kne−e=∫BZdk’2πd∑n’∑GG’ρ~knk’n’Gρ~knk’n’*G’   ×4πe2k’−k+G2Im⁡εGG’−1k’−k,Ekn−Ek’n’

where ${\tilde{\rho }}_{knk’n’}(G)$ are density matrices expressed in the plane-wave basis, and $\varepsilon_{GG^{’}}^{-1}$ is the RPA dielectric matrix for reciprocal lattice vectors G and $G^{’}$.

To evaluate the impact of electron-phonon scattering, the imaginary part of the quasiparticle self-energy is calculated based on Fermi’s golden rule [48,49]:

(8) Im⁡∑kne−ph=∑n’α∫BZΩdk’2πdgk+qn’,knqα2Im⁡nq,α+1−fkn’Ekn−Ek+qn’−ℏωq,α−iη−nq,α+fkn’Ekn−E(k+q)n’+ℏωq,α−iη

where η is a small Lorentzian broadening considering the effect of thermal oscillation (η=25 meV) [50]. The dependence on ambient temperature is reflected in the occupation numbers of electronic and phononic states. The same quantum states kn used for electron-electron scattering calculations are employed in the integration and summation calculations of Equation (8) while Monte Carlo sampling was used to obtain 7 × 10^4^ sampling points k in the first Brillouin zone to ensure the convergence of the calculation results.

By considering the combined contributions of electron-electron and electron-phonon scattering, the lifetime of the hot carriers is calculated as:

(9) τkn=ℏ2Im⁡∑kne−e+Im⁡∑kne−ph

while the mean free path is calculated as $\lambda_{kn}=\nu_{kn}\cdot \tau_{kn}$, where $\tau_{kn}$ and $\nu_{kn}$ are lifetime and group velocity of the carrier in the electronic state kn, respectively.

## 3. Result and Discussion

### 3.1. Structure and Stability

**Figure 1:**
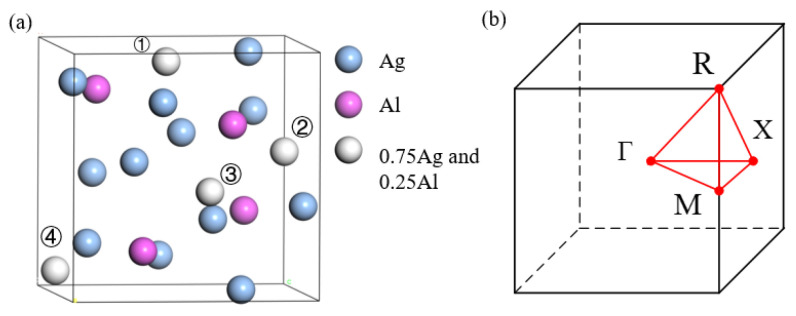
(**a**) The crystal structure of μ-Ag_3_Al, and ①, ②, ③, and ④ represent the four inequivalent positions that Al can occupy. (**b**) The first Brillouin zone and the irreducible k-points of μ-Ag_3_Al.

μ-Ag_3_Al has a simple cubic structure with a space group of P213 and a Pearson symbol of cP20 [34,51]. Figure 1a shows the crystal structure of μ-Ag_3_Al, and the lattice constants are a=b=c=6.942 Å.

**Figure 2:**
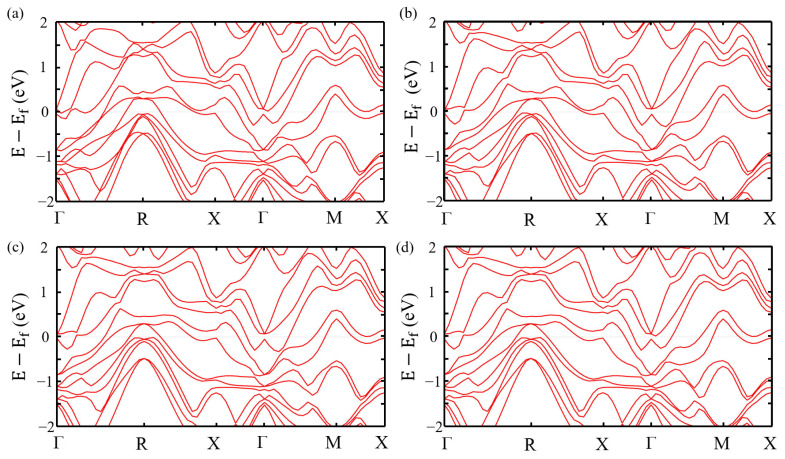
The band structures of μ-Ag_3_Al, with Al atoms occupying four different positions. They are calculated along the irreducible k-points path Γ-R-X-Γ-M-X. (**a**) Al at ①. (**b**) Al at ②. (**c**) Al at ③. (**d**) Al at ④.

As shown in Figure 1a, Al atoms can occupy four inequivalent positions, represented by light gray spheres. Figure 2 shows the band structure of μ-Ag_3_Al with Al atoms occupying the four different atomic positions. Although the atomic positions are not identical, the band structures are almost the same, with only minor differences in a few areas. And the difference in the total energies among the four systems are found to be within 10^−8^ eV. Considering that the differences in the band structure and total energies among the four are negligible, we chose one for further analysis. Additionally, the band structure of μ-Ag_3_Al is very dense, which significantly increases the probability of direct interband transitions of electrons. This also allows direct interband transitions to occur at very low incident light energies, even continuously from near 0 eV.

**Figure 3:**
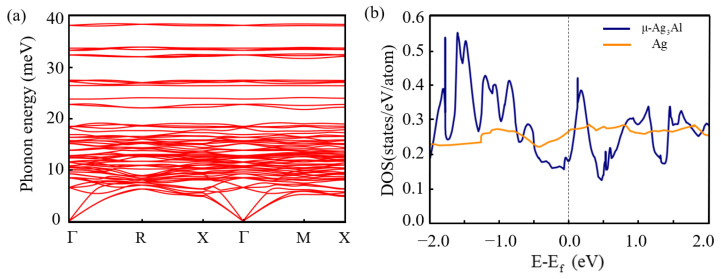
(**a**) Phonon dispersion of μ-Ag_3_Al. (**b**) Total density of states of μ-Ag_3_Al and Ag.

Figure 3a shows the phonon dispersion of μ-Ag_3_Al. All the phonon modes have no imaginary frequencies, indicating the dynamic stability of μ-Ag_3_Al. Figure 3b shows total the density of states (TDOS) of μ-Ag_3_Al and Ag near the Fermi level. The TDOS of μ-Ag_3_Al is higher than that of Ag at most energy levels near the Fermi level, suggesting that the carrier density of μ-Ag_3_Al is higher than that of Ag. Meanwhile, the introduction of Al results in an asymmetric TDOS for μ-Ag_3_Al with respect to the Fermi level, which may lead to an asymmetric distribution of hot electrons and hot holes in μ-Ag_3_Al [52].

### 3.2. Optical Properties

**Figure 4:**
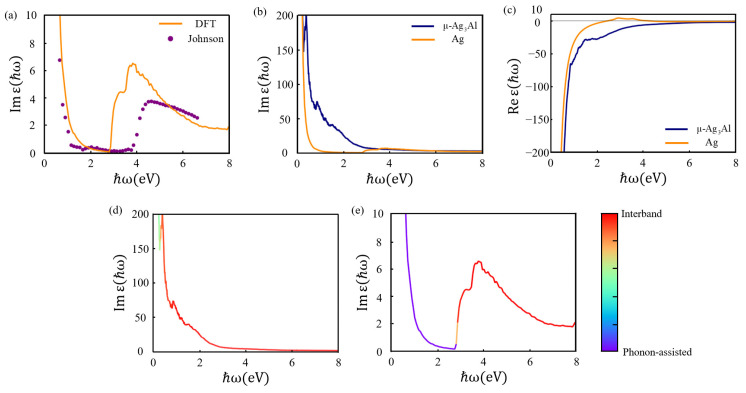
(**a**) Imaginary part of the dielectric function of Ag obtained by experimental measurement and our calculation. (**b**) Imaginary part of the dielectric function of μ-Ag_3_Al and Ag. (**c**) Real part of the dielectric function of μ-Ag_3_Al and Ag. (**d**,**e**) Contributions of direct interband and phonon-assisted intraband in μ-Ag_3_Al and Ag.

The dielectric function of any material is a complex function, denoted as $\varepsilon \left(\omega \right)=\varepsilon_{1}\left(\omega \right)+i\varepsilon_{2}\left(\omega \right)$. In order to verify the accuracy of our theoretical methods, the dielectric function of Ag was calculated. As shown in Figure 4a, the result is very close to the experimental values in [53] below the interband threshold. Above the interband threshold, the result exhibits a slight red-shifted relative to the experimental results. This is because the used PBE functional underestimates the energy of the interband transition. Figure 4b shows the imaginary part of the dielectric functions $\varepsilon_{2}\left(\omega \right)$ of μ-Ag_3_Al and Ag. In the range of 0–3 eV, $\varepsilon_{2}\left(\omega \right)$ of μ-Ag_3_Al is always greater than that of Ag. This enhancement is attributed to the introduction of Al, which brings in impurity-induced states and consequently increases electron scattering. Meanwhile, a larger value of $\varepsilon_{2}\left(\omega \right)$ is more favorable for nanophotonic applications [54]. As shown in Figure 4c, the real part of the dielectric function $\varepsilon_{1}(\omega )$ of μ-Ag_3_Al is negative in the 0–8 eV range. From the electromagnetic wave vector equation $\omega^{2}\varepsilon =c^{2}\left(k\cdot k\right)$, for a physically real frequency ω, $\varepsilon_{1}(\omega )<0$ implies that the electromagnetic wave vector k is imaginary. This means that electromagnetic waves in the corresponding band cannot propagate in μ-Ag_3_Al. $\varepsilon_{1}(\omega )$ of Ag changes from negative to positive around 2.8 eV, allowing electromagnetic waves to propagate above 2.8 eV in Ag.

Figure 4d,e show the contributions of direct interband and phonon-assisted intraband electronic transitions to $\varepsilon_{2}\left(\omega \right)$. The color scale represents the normalized relative contribution ratio. For μ-Ag_3_Al, direct interband transition is the dominant physical mechanism across nearly all wavelength ranges. This is due to the introduction of Al, which modifies the alloy’s d-band and enhances the interband transitions [52]. Whereas the dominant mechanism in the 0–3.4 eV range is phonon-assisted interband transition for Ag, and direct interband electronic transitions become dominant above 3.4 eV.

### 3.3. Plasmon Properties

The energy loss function describes the energy loss of high kinetic energy electrons traversing a homogeneous material, which is calculated as follows [55].

(10) Lω=Im⁡−1εω=ε2ωε12ω+ε22ω

**Figure 5:**
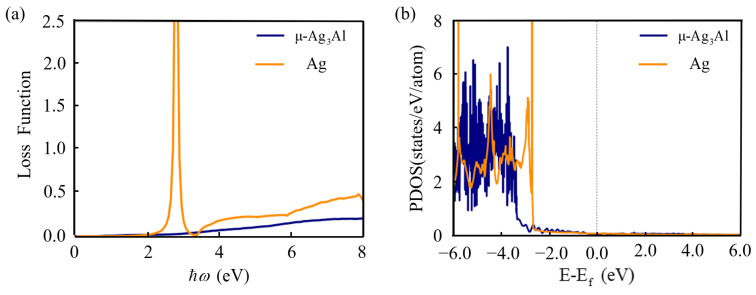
The energy loss function (**a**) and orbital projected density of states (**b**) of μ-Ag_3_Al and Ag.

The peak of the loss function represents the characteristics related to collective oscillations of electrons, which is called bulk plasmon, and the corresponding frequency is called the bulk plasmon frequency $\omega_{p}$. As shown in Figure 5a, μ-Ag_3_Al exhibits no distinct peak. Ag generates a peak near 2.8 eV, which is close to the zero point of $\varepsilon_{1}(\omega )$. The difference of the $\omega_{p}$ between calculated result and established experimental values is because the first-principles calculations underestimate the energy of d-band electrons. As shown in Figure 5b, the orbital projected density of states (PDOS) of d-band electrons of Ag are mainly concentrated below −2.8 eV, which is in agreement with the previous report [56]. That is 1 eV lower than the energy of the d-band electrons from experiments. The PDOS of d-bands of μ-Ag_3_Al are concentrated below −3.5 eV and the hot carriers discussed later are not within this range. Therefore, the results should be reasonable. At this frequency, there is significant electron energy loss in Ag. Overall, within the range of 0–8 eV, the energy loss of electrons in μ-Ag_3_Al is less than that in Ag.

**Figure 6:**
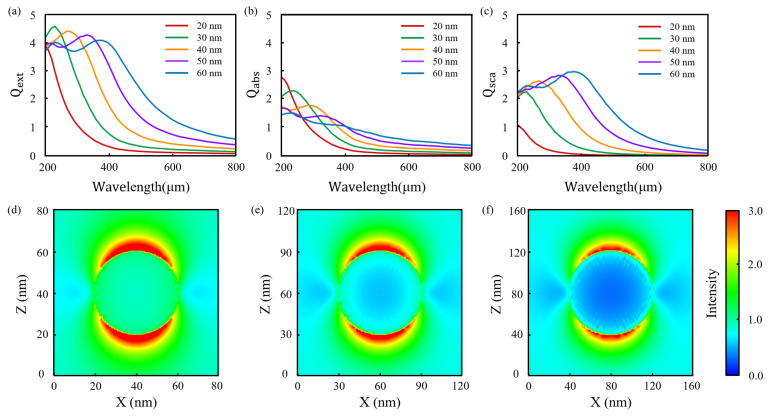
The scattering and absorption properties of μ-Ag_3_Al (**a**–**c**) and near-field simulations of μ-Ag_3_Al nanoparticles with radii of 20 nm, 30 nm and 40 nm (**d**–**f**).

Surface plasmons, primarily classified as localized surface plasmon resonance (LSPR) and surface plasmon polaritons (SPP), are widely used in practical photoconversion applications. Nanoparticles are one of the most important platforms for exciting LSPR. The discrete dipole approximation (DDA) method is employed to simulate the scattering and absorption properties of μ-Ag_3_Al spherical nanoparticles with diameters of 20 nm, 30 nm, 40 nm, 50 nm, and 60 nm, in order to investigate their localized surface plasmon resonance LSPR behavior. We performed the simulations using 5.12 × 10^5^ dipoles, and the results were convergent. As shown in Figure 6a–c, the absorption and scattering properties of nanoparticles vary significantly with changes in radius. As the radius increases, the peak wavelengths of $Q_{ext}$, $Q_{abs}$ and $Q_{sca}$ for μ-Ag3Al exhibit a red shift. When the radius changes, the peak value of $Q_{ext}$ remains relatively stable, while $Q_{abs}$ decreases and $Q_{sca}$ increases with the radius. This indicates that for smaller radii, μ-Ag_3_Al primarily exhibits absorption characteristics, whereas for larger radii, scattering becomes the dominant feature. Moreover, the peak values of $Q_{ext}$ of all four are greater than 3.5, suggesting the presence of a pronounced LSPR at the peak wavelength. Figure 6d,e present the near-field distributions of nanoparticles with radii of 20 nm, 30 nm, and 40 nm, which the simulated wavelengths are 200 nm, 230 nm and 270 nm, respectively. All three exhibit a strong LSPR effect. The above results suggest that μ-Ag_3_Al exhibits strong potential for applications in plasmon-based ultraviolet photoconversion.

In practical photoconversion applications, surface plasmon is widely used. In the following, we primarily focus on surface plasmon polaritons (SPP). SPP is electromagnetic mode excited by the coupling of electromagnetic fields with the collective oscillations of electrons at the surface of metals. The simplest geometry sustaining SPP is a single, flat interface between a dielectric half space and a conducting half space. The spatial characteristics of SPP stems from the features of their dispersion relation, which links the allowed frequencies of surface plasmon oscillations with their propagation wave vectors. The dispersion relation of SPP can be expressed as follows [12,57,58,59]:

(11) $$\beta =k_{0}\sqrt{\frac{\varepsilon_{r}\varepsilon \left(\omega \right)}{\varepsilon_{r}+\varepsilon \left(\omega \right)}}$$

where $k_{0}=\frac{\omega }{c}$ is the wave vector of electromagnetic waves in vacuum; c is the speed of light in vacuum; $\varepsilon \left(\omega \right)$ is the complex dielectric function of the metal under study; and $\varepsilon_{r}$ is the dielectric constant of the dielectric medium.

Silicon has achieved significant accomplishments in both theoretical research and commercial applications in optoelectronic devices. However, its transparency covers only the infrared wavelength range, specifically from 1.1 μm to 4 μm. In contrast, silicon nitride spans a broader operational range from visible light to mid-infrared wavelengths, ranging from 400 nm to 4 μm [60,61,62]. Therefore, we select material Si_3_N_4_ as the dielectric in the aforementioned structure supporting SPP, setting $\varepsilon_{r}=5$ [61]. When $\varepsilon_{r}+\varepsilon \left(\omega \right)=0$, i.e., $\varepsilon \left(\omega \right)=-5$, $\beta \gg k_{0}$ and the group velocity approaches zero. Under these conditions, the SPP mode exhibits electrostatic field characteristics, which is referred to as a surface plasmon. The corresponding frequency is known as the surface plasmon frequency $\omega_{sp}$. From the previous calculations of the dielectric function, it is known that the $\omega_{sp}$ of Ag is about 1.95 eV, and $\omega_{sp}$ of μ-Ag_3_Al is about 4.8 eV. When the frequency of the incident light is less than $\omega_{sp}$, the electromagnetic waves are confined near the interface, and the SPP is in a bound or non-radiative mode. Conversely, when the frequency of the incident light is greater than $\omega_{sp}$, the electromagnetic waves radiate into the metal layer, and the SPP is in a radiative mode [11,12].

**Figure 7:**
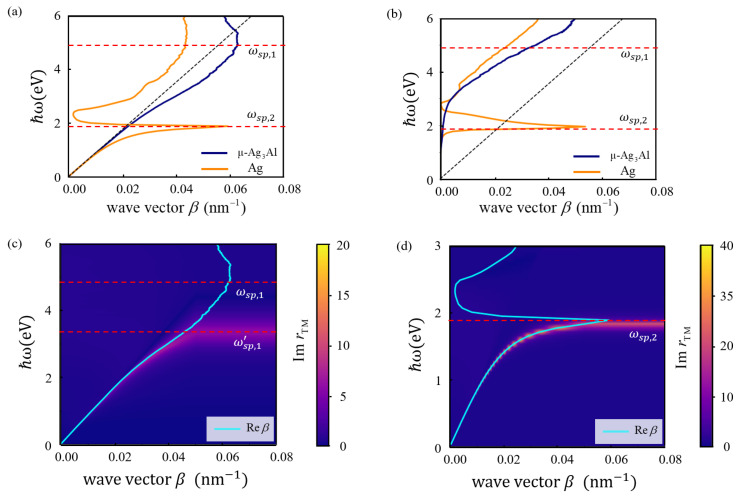
The SPP dispersions of μ-Ag_3_Al/ Si_3_N_4_ and Ag/Si_3_N_4_. (**a**) Real part of wave vector Re⁡β. (**b**) Imaginary part of wave vector Im⁡β. (**c**,**d**) The peak of $Imr_{TM}$ at the interface of μ-Ag_3_Al/ Si_3_N_4_ and Ag/Si_3_N_4_. The red dashed line represents the plasmon resonance frequency of the material under different analytical conditions.

The SPP dispersion relations of μ-Ag_3_Al/Si_3_N_4_ and Ag/Si_3_N_4_ are shown in Figure 7a,b, where the vertical axis represents the frequency of the incident light, and the horizontal axis represents the wave vector of the SPP. $\omega_{sp,1}$ and $\omega_{sp,2}$ are the surface plasmon frequencies of μ-Ag_3_Al and Ag, respectively. The black dashed line represents the wave vector of light propagating in the Si_3_N_4_ medium. Due to the inherent confinement properties of SPP, when the incident light frequency is less than $\omega_{sp}$, the real part of the SPP wave vector Re⁡β is greater than the wave vector of light in Si_3_N_4_. The imaginary part of the SPP wave vector Im⁡β represents the dissipation of SPP. As shown in Figure 7a, when the SPPs of both μ-Ag_3_Al and Ag are in non-radiative modes, Re⁡β gradually increase and deviate from the black dashed line as the energy of the incident light increases, reaching peaks at $\omega_{sp}$. The deviation of Re⁡β of Ag from the black dashed line is significantly greater than that of μ-Ag_3_Al, indicating that Ag exhibits a superior localization effect for electromagnetic wave compared to μ-Ag_3_Al. When the energy of the incident light exceeds $\omega_{sp}$, Re⁡β of both gradually decrease and are positioned to the left of the black dashed line, indicating that the SPPs enter the radiative mode. As shown in Figure 7b, when the energy of the incident light is low, the loss of both SPP is similar. When the energy of the incident light exceeds 3 eV, the SPP of μ-Ag_3_Al remains in the non-radiative mode, while the SPP of Ag has entered the radiative mode. However, the losses of the former are greater than those of the latter, indicating that the SPPs of μ-Ag_3_Al exhibit a very strong non-radiative decay currently, suggesting efficient generation of plasmon-induced hot carriers.

The dispersion of SPP coincides with the peak of the imaginary part of the reflection coefficient of the transverse magnetic (TM) electromagnetic mode $Imr_{TM}$. $Imr_{TM}$ at the interfaces of μ-Ag_3_Al/Si_3_N_4_ and Ag/Si_3_N_4_ are shown in Figure 7c,d. The appearance of the peak of $Imr_{TM}$ indicates that the energy of the reflected electromagnetic waves is reduced, which is due to the destructive interference between the reflected electromagnetic waves and SPP [63,64]. When the frequency of the incident light is relatively low, the propagation wave vector of the SPP matches well with the peak of the imaginary part of the reflection coefficient. When the incident light frequency exceeds $\omega_{sp}$, the peak is not observed. This is because SPP transitions to a radiative mode, preventing the electromagnetic wave energy from localizing near the interface. Consequently, the destructive interference between the reflected electromagnetic waves and SPP becomes negligible. As shown in Figure 7c, $Imr_{TM}$ of μ-Ag_3_Al no longer exhibits peaks above 3.36 eV, which we define as $\omega_{sp,1}^{’}$. As shown in Figure 7d, the agreement between Re⁡β and $Imr_{TM}$ is excellent, indicating that the non-radiative decay of the SPPs at Ag/Si_3_N_4_ interface is relatively small. From another perspective, the peak value of $Imr_{TM}$ at the interface Ag/ Si_3_N_4_ is twice that of μ-Ag_3_Al, indicating that the SPP energy at the Ag/Si_3_N_4_ interface is very strong. This strong contract highlights the significant non-radiative loss of SPP at the μ-Ag_3_Al/Si_3_N_4_ interface. In conclusion, the pronounced peak of $Imr_{TM}$ of μ-Ag3Al appears at energies ranging from 0 eV to 3.36 eV, and that of Ag appears from 0 eV to 1.96 eV, indicating the surface plasmon operating bandwidth of μ-Ag_3_Al is 1.7 times that of Ag.

### 3.4. Hot Carriers

Extensive experiments and research have shown that the non-radiative decay of SPP generates a large number of hot carriers (hot electrons and hot holes), which have great potential in the field of plasmonic optoelectronics. In practical applications, the energy distribution of hot carriers plays a crucial role in their collection and utilization efficiency. The dominant factor for hot carrier generation in μ-Ag_3_Al is interband direct transitions, while phonon-assisted intraband transitions are second-order processes and are generally weak. Therefore, the properties of hot carriers originating from interband direct transitions in μ-Ag_3_Al and Ag are provided.

**Figure 8:**
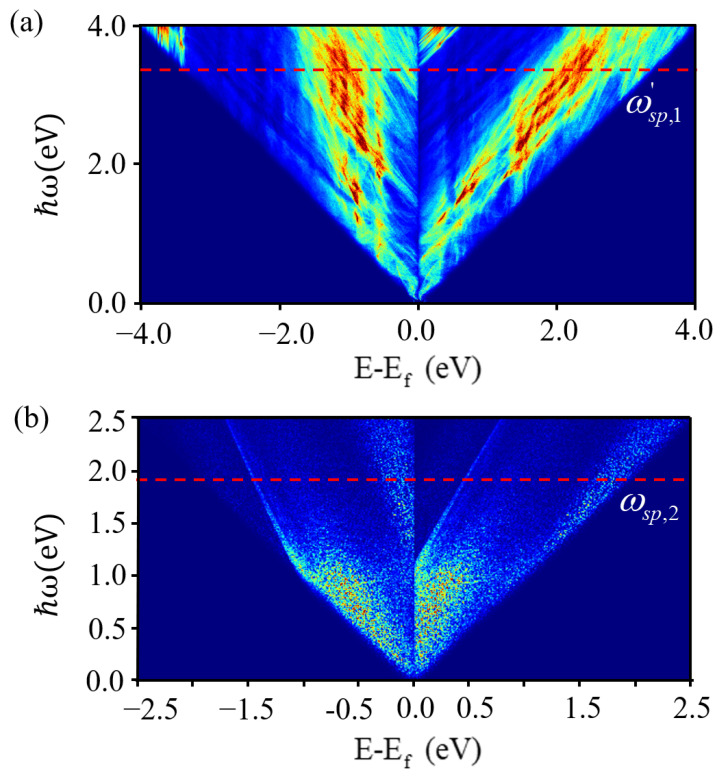
Energy distribution of hot carriers generated by direct interband transitions. (**a**) μ-Ag_3_Al. (**b**) Ag. The red dashed line represents the plasmon resonance frequency of the material.

As shown in Figure 8, the horizontal axis represents the energy of hot carriers, with negative values indicating hot holes and positive values indicating hot electrons. The vertical axis represents the incident light energy, and the color scale indicates the generation efficiency of hot carriers. As shown in Figure 8a, When the incident light energy is less than $\omega_{sp,1}^{’}$, μ-Ag_3_Al can generate a significant number of hot carriers, with the energy of hot electrons being higher than that of hot holes. The asymmetric distribution of hot carriers is attributed to the introduction of Al. And the continuous energy distribution in hot carriers facilitates their collection and utilization [22]. As a result, interband transitions within μ-Ag_3_Al are highly prone to take place. This characteristic is of great advantage for its application in the photoelectric conversion. As shown in Figure 8b, when the incident light energy is greater than $\omega_{sp,2}$, the generation of hot carriers in Ag is rather small. In general, hot electrons are easier to measure than hot holes, and hot carriers with higher energy are more easily collected and applied [3,65,66], therefore, μ-Ag_3_Al is more suitable than Ag for constructing plasmon induced hot carriers based optoelectronic devices. Moreover, the energy of hot electrons in μ-Ag_3_Al is generally more than 1eV above the Fermi level and the distribution in the energy space is continuous. In summary, the plasmon-induced high-energy hot electrons generation efficiency of μ-Ag_3_Al is superior to that of commonly studied plasmonic materials such as Au, Ag, and Cu [22,55].

**Figure 9:**
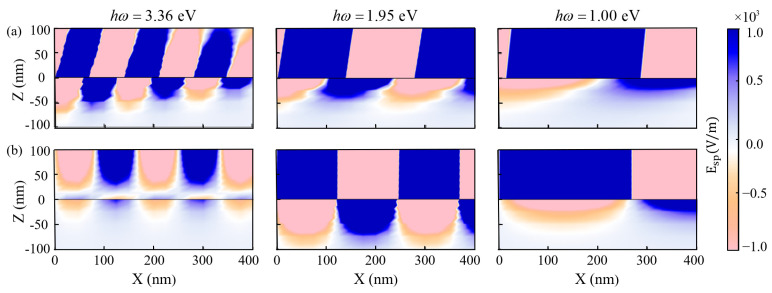
The electric field distribution of SPPs at interfaces of (**a**) μ-Ag_3_Al/ Si_3_N_4_ and (**b**) Ag/Si_3_N_4_.

To further reflect the capability of generating hot carriers by SPPs at interfaces of μ-Ag_3_Al/ Si_3_N_4_ and Ag/Si_3_N_4_, we simulated their electric field distribution in COMSOL Multiphysics 6.0. A computational mesh comprising $1.6\times 10^{5}$ uniformly sized was established across a rectangular domain measuring 2.2 μm in the Z-direction and 10 μm in the X-direction. The excitation geometry is characterized by an electromagnetic wave propagating along the positive X-direction and the port input power density is set to 1 W/m. As shown from left to right in Figure 9, the incident light energies are respectively 3.36 eV, 1.95 eV, and 1.00 eV. The region with Z>0 represents Si_3_N_4_, while the region with Z<0 represents μ-Ag_3_Al or Ag. SPPs propagate in the positive X direction. The Color bar represents the electric field intensity values. A greater penetration depth of the SPP electric field in the negative Z direction indicates a stronger interaction between the light and μ-Ag_3_Al or Ag, which is more beneficial to generating hot carriers [3]. As the SPP propagates, the penetration depth in μ-Ag_3_Al decreases, while that in Ag remains almost unchanged, further indicating that the SPP loss is greater at μ-Ag_3_Al/ Si_3_N_4_ interface than at Ag/ Si_3_N_4_ interface. This can likely be ascribed to the excitation of an extensive number of hot carriers by SPPs. When the incident light energy is 3.36 eV, the maximum penetration depth of in μ-Ag_3_Al is about 70 nm. Meanwhile, the electric field is almost absent near the Ag/ Si_3_N_4_ interface, possibly due to the fact that 3.36 eV is greater than the plasmon frequency 2.8 eV of Ag, leading to light propagation within Ag and the electric field cannot be localized near the interface. When the incident light energy is 1.95 eV, the maximum penetration depth in μ-Ag_3_Al is about 50 nm and that in Ag is about 90 nm. When the incident light energy is 1.00 eV, the maximum penetration depths in both are about 50 nm. Overall, the μ-Ag_3_Al/ Si_3_N_4_ interface can more efficiently generate hot carriers through SPP from UV to near-infrared range.

**Figure 10:**
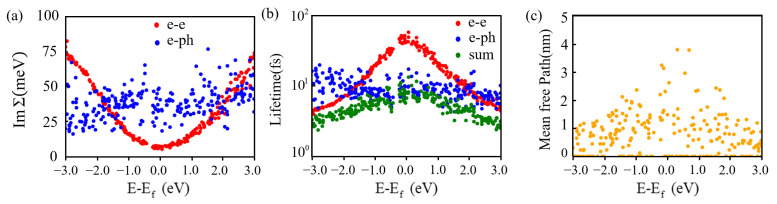
Scattering rate (**a**), lifetime (**b**), and mean free path (**c**) of μ-Ag_3_Al.

In experiment, hot carriers must survive for a sufficiently long time and travel a sufficient distance to be effectively collected or detected. As shown in Figure 10, we respectively present the scattering rates (represented by the imaginary part of the self-energy), lifetimes, and mean free paths of hot carriers at different energy levels of μ-Ag_3_Al. As expected, the electron-electron scattering rate shown in Figure 10a exhibits a parabolic shape, with a minimum at the Fermi level and dominate at regions far from the Fermi level, consistent with the predictions of Fermi liquid theory [67]. And the large electron-electron scattering rates are mainly due to the interband impact ionization and Auger recombination processes. On the other hand, the electron-phonon scattering rates are significantly larger than the electron-electron scattering rates at energies near the Fermi level [22]. Moreover, the dependence of electron-phonon scattering rates on energy is generally consistent with the corresponding electronic density of states shown in Figure 3b. Simultaneously, the electron-phonon scattering rate is similar at different energy levels, indicating that electron-phonon scattering dominates near the Fermi level. Overall, the total lifetime of μ-Ag_3_Al is symmetric with respect to the Fermi level, which is different from that of Al and Ag. μ-Ag_3_Al exhibits a maximum lifetime of about 10 fs near the Fermi level, similar to Al [38]. As shown in Figure 10c, the mean free path of hot carriers is within 4 nm, which is close to that of borophene and Au-Al intermetallic compounds [22,40]. Particularly, hot carriers at energy levels of −3 and 3 eV maintain the mean free path of about 1 nm, which is significant for applications of μ-Ag_3_Al based on hot carriers.

The transport properties of hot carriers at different energy levels are summarized in Table 1.

## 4. Conclusions

In summary, we systematically investigated the optical properties, plasmons, and hot carrier-related properties of μ-Ag_3_Al through the first-principles calculation. Due to the dense energy bands, direct interband electronic transitions in μ-Ag_3_Al can occur over almost any wavelength range. Spherical nanoparticles of μ-Ag_3_Al are capable of supporting LSPR in the ultraviolet region. By analyzing the SPP dispersion relation and the peak values of $Imr_{TM}$, we found that SPP at the μ-Ag_3_Al/Si_3_N_4_ interface exhibits very strong non-radiative decay, which means strong light-matter interactions occur. This suggests that we should pay attention to non-radiative decay wavebands when studying the properties of plasmon. Through the analysis of the energy distribution of hot carriers and the SPP electric field distribution, μ-Ag_3_Al is capable of efficient generating hot carriers from UV to near-infrared. Moreover, the maximum lifetime of the hot carriers is comparable with that of Al, reaching up to 10 fs. High-energy hot electrons and hot holes can simultaneously maintain the mean free path of 1 nm. Therefore, μ-Ag_3_Al should be an alternative plasmonic material for plasmon-induced hot carrier applications from UV to near infrared range, which is helpful to the development of photovoltaics, photodetection, photochemistry, and so on. For example, μ-Ag_3_Al could be considered as an alternative material for broadband photodetector applications because of its dense energy bands and broadband plasmon operating bandwidth [68,69].

## Figures and Tables

**Table 1:** Lifetime and mean free path of hot carriers at different energy levels in μ-Ag_3_Al.

Energy Level eV	Lifetime fs	Mean Free Path nm
−3.0	3.2	1.04
−2.0	4.2	1.39
−1.0	6.7	1.96
0.0	9.8	3.47
1.0	6.6	2.41
2.0	4.3	1.56
3.0	3.0	1.08

## Data Availability

Data underlying the results presented in this paper are not publicly available at this time but may be obtained from the authors upon reasonable request.
